# Association between atrial fibrillation and age-related macular degeneration: A nationwide cohort study

**DOI:** 10.1038/s41433-025-03956-2

**Published:** 2025-09-05

**Authors:** Hou-Ren Tsai, Wei-Chuan Chang, Yuan-Chieh Lee

**Affiliations:** 1 Department of Ophthalmology, Hualien Tzu Chi Hospital, Buddhist Tzu Chi Medical Foundation, Hualien, Taiwan; 2 School of Medicine, Tzu Chi University, Hualien, Taiwan; 3 Epidemiology and Biostatistics Center, Hualien Tzu Chi Hospital, Buddhist Tzu Chi Medical Foundation, Hualien, Taiwan; 4 Department of Ophthalmology and Visual Science, Tzu Chi University, Hualien, Taiwan

**Keywords:** Macular degeneration, Epidemiology

## Abstract

**Background/Objectives:**

Atrial fibrillation (AF) and age-related macular degeneration (AMD) are common debilitating conditions that share pathomechanisms involving chronic inflammation and oxidative stress. However, the association between AMD and AF, which is important for comprehending the pathogenesis, referral, and treatment strategies of these diseases, remains unknown.

**Subjects/Methods:**

This nationwide population-based retrospective cohort study used claims data from the National Health Insurance Research Database of Taiwan. From 1 January 2003 to 31 December 2018, an AF and AMD cohort of 34,236 and 31,766 patients, respectively, along with their matched control cohorts, were constructed using stabilised inverse probability of treatment weighting. The primary outcomes were the incidence of AMD following newly diagnosed AF and the incidence of AF following newly diagnosed AMD. A Cox regression model was used to estimate hazard ratios (HR) for these outcomes. Subgroup analysis based on AMD subtype and stratified analyses by age and sex were also conducted.

**Results:**

Compared with controls, patients with AF had a significantly increased risk of developing AMD (HR 1.10; 95% confidence interval [CI] 1.04–1.17). Similarly, patients with AMD had a significantly increased risk of developing AF (HR 1.08; 95%CI 1.02–1.15). Further subgroup analysis revealed a reciprocal association between AF and dry AMD. Age- and sex-stratified analyses demonstrated increasing risk trends for AF and AMD.

**Conclusions:**

AF and AMD may share common underlying risk factors and pathophysiological mechanisms. Our findings identified a reciprocal association between AF and AMD. Further research is warranted to elucidate the pathophysiology of AF and AMD.

## Introduction

Age-related macular degeneration (AMD) epitomises a significant cause of visual impairment, constituting 8.7% of blindness cases globally and emerging as the predominant cause of blindness in developed nations, particularly among individuals over 60 years old [1, 2]. The exact mechanism of AMD remains elusive, although chronic inflammation, complement activation, and oxidative stress are suggested as potential factors [3]. Various cardiovascular diseases (CVDs), such as coronary artery disease, stroke, and heart failure, have been linked with AMD [4–6]. Atrial fibrillation (AF) is the most common clinically significant cardiac arrhythmia and presents an important public health challenge in the global aging population [7]. The overall predominance of AF ranges from approximately 0.5–4%, heightening with age to almost 3.7–4.2% among individuals aged 60–70 years and 10–17% among those aged ≥80 years [8, 9]. Previous studies have documented the role of inflammation, oxidative stress, and vascular endothelial growth factor (VEGF) in AF, all of which are implicated in the pathogenesis of AMD [10–12]. Abnormal angiogenesis and the components of Virchow’s triad for thrombogenesis, which are hallmark features of AF, have also been associated with AMD, indicating shared pathological mechanisms between the two conditions [13]. Additionally, AF and AMD share environmental risk factors, including cigarette smoking, physical activity, systemic hypertension, and hyperglycaemia [3, 14–18]. Only one cross-sectional study with a small sample size reported no independent association between AMD and AF prevalence [19]. However, given the shared pathogenetic and risk factors of these two diseases, one may promote the subsequent development of the other.

Therefore, we investigated the reciprocal association between AMD and AF. We hypothesised the existence of an association between AMD and AF and conducted a nationwide population-based cohort study to evaluate the risk of developing AMD or AF in patients with newly diagnosed diseases.

## Materials and methods

### Data source

This cohort study used data from the National Health Insurance Research Database (NHIRD) of Taiwan containing claims information. The National Health Insurance program of Taiwan, introduced in 1995, is a compulsory single-payer healthcare system operated by the government, covering over 99.6% of the Taiwanese population [20, 21]. The NHIRD provides anonymised data from the National Health Insurance program for research, offering healthcare details to approximately 23.6 million insured individuals, encompassing the entire population of Taiwan [20, 21]. This database encompasses comprehensive information on all enrolled individuals, including demographic data, healthcare claims, and medication records. Diagnosis and procedure codes were sourced from the International Classification of Diseases, Ninth Revision, Clinical Modification (ICD-9-CM) until 2016, and subsequently from the Tenth Revision (ICD-10-CM). This study used the Longitudinal Health Insurance Database, a subset of the NHIRD, containing data from a random sample of 2 million patients from 2000 to 2019. Epidemiological studies have validated the Longitudinal Health Insurance Database as a precise representation of the general Taiwanese population [22].

### Study population in the NHIRD

Between 1 January 2003 and 31 December 2018, adults aged ≥50 years, who contributed to the NHIRD, were enrolled. We first identified an exposed (AMD) cohort comprising individuals with newly diagnosed AMD and an unexposed (non-AMD) cohort involving individuals without an AMD diagnosis during the entire study period to evaluate the bidirectional association between AMD and AF. AMD definition cases required a diagnosis confirmed by at least two separate outpatient department diagnoses or via a discharge diagnosis by ophthalmologists using ICD-9-CM codes 362.50, 362.51, 362.52, and 362.57, and ICD-10-CM codes H35.30, H35.31, H35.32, and H35.36. These diagnostic codes, previously employed in other studies, appear to demonstrate high positive predictive values [23, 24]. The index date for patients in the AMD cohort was defined as the first outpatient appointment date or inpatient AMD diagnosis, whichever occurred earlier. The index date for each patient with non-AMD was assigned to match the index date as their matched patient with AMD. Patients with AMD records prior to the specified index dates were excluded from both cohorts.

We included an exposed (AF) cohort comprising individuals with newly diagnosed AF and an unexposed (non-AF) cohort consisting of individuals without any AF diagnosis. The definition of AF cases entailed an AF diagnosis authenticated by at least two separate outpatient department diagnoses or via a discharge diagnosis using ICD-9-CM code 427.31 and ICD-10-CM codes I48.0, I48.1, I48.2, and I48.91; using Taiwanese NHIRD, these diagnostic codes were validated [25]. The AF cohort index dates were described as the date of first outpatient visit or inpatient AF diagnosis, whichever occurred first. The index date for the non-AF group was designated to match the index date of the matched AF group. Patients with any AF record before the respective index dates were excluded from both cohorts.

### Outcome measures

The primary outcome was the occurrence of incident AF (ICD-9-CM code 427.31 and ICD-10-CM codes I48.0, I48.1, I48.2, and I48.91) in the AMD cohort. Similarly, the occurrence of incident AMD, including diagnoses of macular degeneration (senile), unspecified (ICD-9-CM codes: 362.50; ICD-10-CM codes: H35.30), nonexudative senile macular degeneration (ICD-9-CM codes: 362.51; ICD-10-CM codes: H35.31), exudative senile macular degeneration (ICD-9-CM codes: 362.52; ICD-10-CM codes: H35.32), and drusen (degenerative) (ICD-9-CM codes: 362.57; ICD-10-CM codes: H35.36) was the primary outcome in the AF cohort. An outcome event was defined as a diagnosis made during outpatient or inpatient services. All study participants were tracked from their respective index dates until the occurrence of outcome events, death, or the last date in the database, whichever occurred first. Additionally, we performed individual analyses of dry AMD (ICD-9-CM codes: 362.50, 362.51, and 362.57; ICD-10-CM codes: H35.30, H35.31, and H35.36) and wet AMD (ICD-9-CM code: 362.52; ICD-10-CM code: H35.32), wherein patients were followed up until the desired outcome occurred. Therefore, a patient with dry AMD who later developed wet AMD was included in the wet AMD analysis.

### Covariates and confounders

Eligible individuals’ baseline characteristics were extracted from the NHIRD. Pre-existing comorbidities and baseline medication use (see Supplementary Table S1) were considered potential confounding factors, guided by precedent research [25–28]. Any pre-existing comorbidity was established as a discharge diagnosis or a diagnosis confirmed ≥ twice in an outpatient setting within the year preceding the index date. The overall comorbidity conditions within each respective group were evaluated using the Charlson Comorbidity Index [29]. Baseline medication use was defined as a prescription drug taken for 30 days or more within the year prior to the index date. Income-related National Health Insurance premiums were employed to ascertain monthly income levels.

### Statistical analyses

Stabilised inverse probability of treatment weighting (IPTW), a propensity score method, was used to address potential systematic differences between the baseline characteristics of the paired groups (AMD versus Non-AMD or AF versus Non-AF) [30]. The variations in baseline characteristics were evaluated using the standardised mean difference (SMD), with a value of less than 0.1 indicating a negligible difference [31]. Additionally, we conducted a sensitivity analysis using propensity-score matching (PSM), performed using the nearest-neighbour matching algorithm without replacement, to balance patient characteristics between groups; a calliper width of 0.2 standard deviations of the logit of the propensity score was employed [32, 33]. The Kaplan–Meier method was used in determining the cumulative incidence of outcome, and log-rank tests were used to evaluate differences between cumulative incidence curves. Cox proportional hazards regression models based on stabilised IPTW estimated the hazard ratio (HR) for each outcome. Statistical significance was set at a *P*-value of *<*.05.

For the AF and AMD cohorts, stratified analyses according to age, sex, and AMD subtype were performed. Subgroup analysis based on the AMD subtype was conducted within the AMD cohort. All statistical analyses were conducted using SAS software (version 9.4; SAS Institute, Cary, NC, USA).

## Results

### Baseline characteristics of the study population

The original population of the AMD and non-AMD cohorts included 63,532 patients (Supplementary Table S2). Following the IPTW approach application, the pseudopopulations comprised 31,726 patients with AMD and 31,997 patients without AMD. All demographic data were properly proportioned between the two study cohorts after IPTW, with all SMDs < 0.1 (Table 1). Supplementary Table S3 outlines the demographic data of the study cohorts after PSM.

**Table 1:** Demographic data of the study population after stabilised inverse probability of treatment weighting.

Characteristics^a^	AMD and the risk of AF			AF and the risk of AMD		
	AMD			AF		
	AMD (*n* = 31,726)	Non-AMD (*n* = 31,997)	SMD^b^	AF (*n* = 33,958)	Non-AF (*n* = 35,827)	SMD^b^
Mean age (SD), y	70.02 (10.0)	70.21 (9.4)	0.020	73.51 (10.7)	74.01 (10.8)	0.047
Sex, %			0.010			0.009
Male	46.8	46.32		54.8	55.2	
Female	53.2	53.7		45.2	44.8	
Income level (NTD), %						
<15840	19.8	17.6	0.056	20.2	21.7	0.038
15,840–24,999	53.6	57.0	0.069	57.1	55.2	0.038
25,000–39,999	13.5	13.4	0.004	11.5	11.9	0.013
≥40,000	13.2	12.1	0.033	11.3	11.2	0.003
CCI, mean (SD)	1.08 (1.7)	1.09 (1.7)	0.006	1.32 (2.0)	1.50 (2.3)	0.082
Comorbidities, %						
Hypertension	32.3	32.2	0.003	35.6	37.1	0.031
Diabetes mellitus	18.7	19.2	0.014	16.8	17.7	0.023
Stroke	7.0	7.0	0.002	12.4	14.5	0.062
Heart failure	2.3	2.3	0.001	10.5	14.0	0.108
Coronary artery disease	10.9	11.0	0.003	17.0	20.2	0.082
Cirrhosis	0.82	0.8	0.004	1.0	1.2	0.016
COPD	7.2	7.2	0.001	10.9	12.7	0.057
Chronic kidney disease	3.5	3.5	0.002	5.3	6.7	0.058
Hyperlipidaemia	20.5	20.7	0.003	16.4	16.8	0.002
Malignancy	4.9	4.9	0.004	5.4	5.7	0.014
Obesity	0.3	0.3	0.004	0.2	0.2	0.004
Cataract	28.8	29.4	0.014	10.4	10.8	0.015
Other concomitant drugs, %						
ACEI/ARB	30.8	31.4	0.013	36.0	38.6	0.053
Beta blocking agents	22.1	22.5	0.011	27.5	29.8	0.050
Calcium channel blockers	31.5	32.0	0.012	37.4	39.0	0.033
Metformin	14.4	15.5	0.028	12.0	12.0	0.001
Statins	19.1	19.6	0.013	15.4	15.7	0.008
Fibrates	3.8	3.9	0.006	3.4	3.5	0.003
Antiplatelets	24.7	25.4	0.017	33.8	37.2	0.071

Data are expressed as percentages unless otherwise indicated.

^a^All covariates listed were used to calculate the propensity score for inverse probability of treatment weighting.

^b^A standardised mean difference of <0.1 indicates a negligible difference.

Angiotensin converting enzyme inhibitor, *ACEI* Atrial fibrillation, *AF* Age-related macular degeneration, *AMD* ARB, Angiotensin II receptor blockers, *CCI* Charlson comorbidity index, *COPD* Chronic obstructive pulmonary disease, *NTD* New Taiwan Dollar, *SD* Standard deviation, *SMD* Standardised mean difference.

Among the AF and non-AF cohorts, the original population comprised 68,472 patients (Supplementary Table S2). After applying the IPTW approach, the pseudopopulation group included 33,958 patients with AF and 35,827 patients without AF. All demographic data were adequately balanced between the two study cohorts following IPTW, with most SMDs < 0.1 (Table 1). Supplementary Table S3 summarises the demographic data of the study cohort after PSM.

### AF risk among patients with AMD

**Fig. 1:**
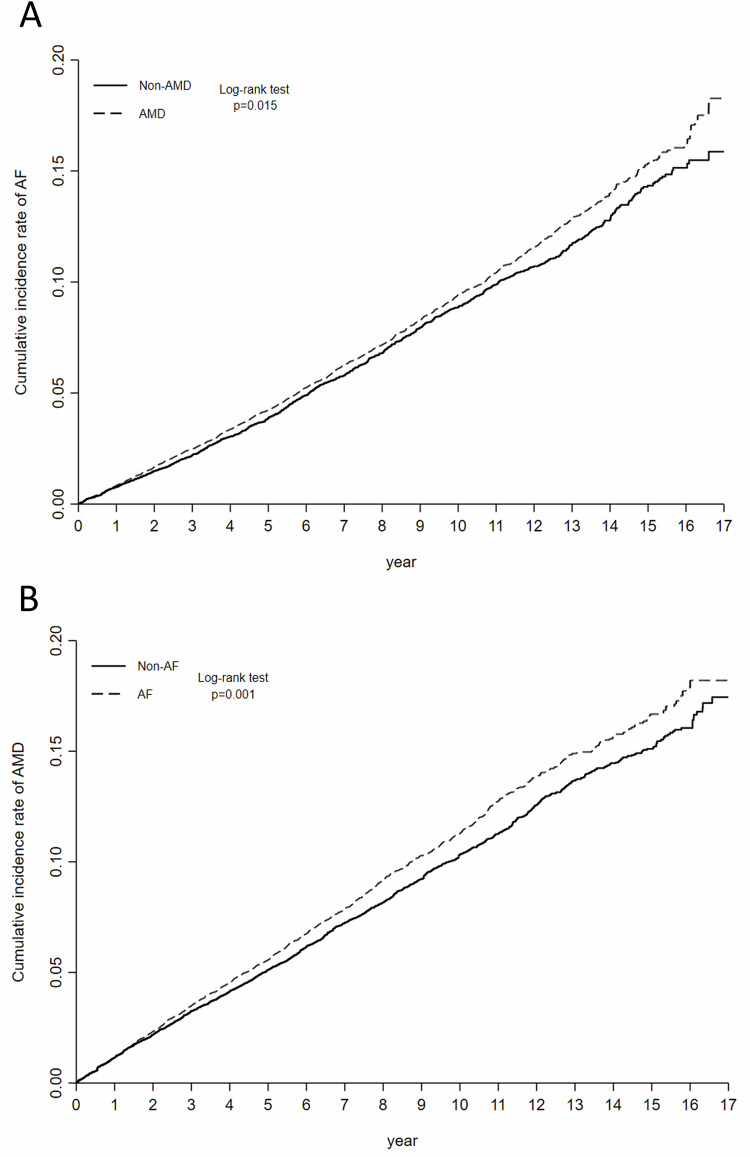
Cumulative incidence curves for outcomes. **A** AMD and the risk of AF and **B** AF and the risk of AMD. *AF* Atrial fibrillation, *AMD* Age-related macular degeneration.

Overall, 2291 patients in the AMD cohort and 1873 in the non-AMD cohort developed AF, with respective incidence rates of 9.85 and 8.95 per 1000 person-years (Table 2). Regarding the AMD and non-AMD cohorts, the mean follow-up periods were 6.40 and 6.46 years, respectively. Patients with AMD were at a heightened risk of incident AF (HR 1.08; 95% confidence interval [CI]: 1.02–1.15; *p* = 0.015) than those without AMD. Figure 1A presents the cumulative incidence curve. Sensitivity analysis using PSM showed consistent results (HR 1.08; 95% CI: 1.01–1.17; *p* = 0.034) (Supplementary Table S4). Supplementary Fig. 1A depicts the PSM cumulative incidence curve.

**Table 2:** Association between AMD and AF.

Comparison/Outcomes	Patients, *n*	Events, *n*	Person-years at risk	Incidence rate^a^	HR^b^	95% CI	*p* value
AMD and the risk of AF							
AMD cohort	31,726	2291	232,716	9.85	1.08	1.02–1.15	0.015
Non-AMD cohort	31,997	1873	209,145	8.95	1.00	Reference	
AF and the risk of AMD							
AF cohort	33,958	2063	172,252	11.98	1.10	1.04–1.17	0.001
Non-AF cohort	35,827	2398	220,314	10.88	1.00	Reference	

^a^Per 1000 person-years.

^b^The hazard ratio was calculated by a univariable Cox regression model considering inverse probability weighting.

*AF* atrial fibrillation, *AMD* age-related macular degeneration, *CI* confidence interval, *HR* hazard ratio, *SD* standard deviation.

### AMD risk among patients with AF

Overall, 2063 patients in the AF cohort and 2398 in the non-AF cohort developed AMD, with incidence rates of 11.98 and 10.88 per 1000 person-years, respectively (Table 2). The mean follow-up periods for the AF and non-AF cohorts were 6.40 and 6.46 years, respectively. Patients with AF were at an elevated risk of incident AMD (HR 1.10; 95% CI: 1.04–1.17; *p* = 0.001) than those without AF. Figure 1B shows the cumulative incidence curve. By applying PSM, the sensitivity analysis conducted showed consistent results (HR 1.09; 95% CI: 1.02–1.17; *p* = 0.014) (Supplementary Table S4). Supplementary Fig. 1B shows the cumulative incidence curve for PSM.

### The association between AMD subtypes and AF

As shown in Table 3, dry AMD was correlated with a higher risk of AF (HR 1.09; 95% CI: 1.02–1.16; *p* = 0.007), whereas patients with wet AMD were less likely to develop AF (HR 0.94; 95% CI: 0.74–1.19; *p* = 0.589) with regard to the individual outcome analyses. Contrastingly, AF was associated with a higher risk of dry AMD (HR 1.12; 95% CI: 1.05–1.19; *p* < .001) but not wet AMD (HR 1.03; 95% CI: 0.84–1.26; *p* = 0.794).

**Table 3:** Association between AMD subtype and AF.

Comparison/Outcomes	Patients, *n*	Events, *n*	Person-years at risk	Incidence rate^a^	HR^b^		95% CI		*p* value
AMD subtype and the risk of AF									
Dry AMD									
Dry AMD cohort	29,865	2157	220,647	9.78	1.09		1.02–1.16		0.007
Non-AMD cohort	30,115	1742	198,112	8.79	1.00		Reference		
Wet AMD									
Wet AMD cohort	1907	140	12,329	11.31	0.94		0.74–1.19		0.589
Non-AMD cohort	1926	134	11,283	11.88	1.00		Reference		
AF and the risk of AMD subtype									
Dry AMD									
AF cohort	33,958	2015	172,424	11.69	1.12		1.05–1.19		<0.001
Non-AF cohort	35,827	2311	220,697	10.47	1.00		Reference		
Wet AMD									
AF cohort	33,958	166	180,904	0.92	1.03		0.84–1.26		0.794
Non-AF cohort	35,827	208	230,467	0.90	1.00		Reference		

^a^Per 1000 person-years.

^b^The hazard ratio was calculated by a univariable Cox regression model considering inverse probability weighting; the hazard ratio is calculated using the Non-AMD or Non-AF cohort as the reference.

*AF* atrial fibrillation, *AMD* age-related macular degeneration, *CI* confidence interval, *HR* hazard ratio, *SD* standard deviation.

### Stratified analysis

The pattern of increased susceptibility to AMD and AF remained consistent in the age- and sex-stratified analyses (Table 4). Conversely, the correlation between AMD and AF was not statistically significant in any female subgroup in the sex-stratified analysis.

**Table 4:** Association between AMD and AF after stratification.

Comparison/Outcomes	HR^‡^	95% CI	*p* value
AMD and the risk of AF			
Sex			
Male	1.12	1.03-1.22	0.010
Female	1.04	0.95-1.13	0.393
Age			
<65 years	1.05	0.89-1.25	0.558
≥65 years	1.10	1.03-1.17	0.006
AF and the risk of AMD			
Sex			
Male	1.13	1.04-1.22	0.003
Female	1.07	0.98-1.17	0.123
Age			
<65 years	1.24	1.08-1.43	0.002
≥65 years	1.11	1.04-1.18	0.003

^‡^The hazard ratio was calculated by a univariable Cox regression model considering inverse probability weighting; the hazard ratio is calculated using the non-AMD or.

Non-AF cohort as the reference.

*AMD* age-related macular degeneration, *AF* atrial fibrillation.

*CI* confidence interval, *HR* hazard ratio, *SD* standard deviation.

## Discussion

In this 20-year longitudinal cohort study, we discovered that compared with the non-AMD cohort, patients with AMD had an 8% increased risk of developing AF. Moreover, patients with AF had a 10% elevated risk of AMD development in comparison with the non-AF cohort. This finding is significant because both AMD and AF are prevalent among older adults, who constitute the fastest-growing demographic group worldwide. The reciprocal relationship between AMD and AF emphasises the significance of early detection of both conditions and the development of prevention and treatment strategies aimed at mitigating the risk of these diseases.

To the best of our knowledge, only a single cross-sectional analysis from the Australian Heart Eye Study (AHES) has investigated the correlation between AMD and AF [19]. In the univariate analysis, this study identified a significant association between early AMD and the predominance of AF, whereas this relationship was no longer significant after the adjustment for potential confounders. However, the cross-sectional design without follow-up data and the limited number of patients with AF (147 patients) and AMD (107 patients) may have restricted its validity. The authors propose that additional longitudinal follow-up research is necessary to authenticate this observation. Our study is the first cohort analysis conducted at a population level to investigate the association between AF and AMD and offers a more advanced level of evidence for causality than the AHES cross-sectional study. Furthermore, the AHES did not examine the risk associated with various AMD subtypes with AF. Our analysis uncovered a small but significant reciprocal association between AF and AMD. Further analysis revealed a significant association between dry AMD but not wet AMD and AF.

Mounting evidence suggests a correlation between AMD and multiple CVDs, such as acute myocardial infarction, stroke, and heart failure [4–6]. Chronic inflammation and oxidative stress form the basis of the connection between AMD and CVDs [34]. Mitigating shared risk factors may aid in AMD and CVDs prevention, emphasising the necessity of prioritising it as a public health concern. Notably, guidelines [35, 36] from Asia have advocated for a holistic or integrated care approach to managing AF. AF is characterised by a multifaceted interplay of inflammation, oxidative stress, and endothelial activation, which shares analogies with AMD [10–12]. Inflammation and its associated immune response are involved in the initiation and maintenance of AF [12]. Additionally, numerous risk factors, such as advanced age, low physical activity, obesity, smoking, and hypertension, have been implicated as risk factors shared between AF and AMD [18, 37]. Abnormal angiogenesis and the elements of Virchow’s triad for thrombogenesis, which are characteristic features of AF, have also been linked to AMD [13]. Therefore, the association between AF and AMD may be explained by the shared underlying risk factors and pathophysiological mechanisms.

AMD is generally divided into two stages: wet and dry at its late stage. In the subgroup and stratified analysis, our findings indicated an association between dry AMD, but not wet AMD, and AF. Moreover, patients with wet AMD had a marginally reduced risk of developing AF. Patients with AF have elevated inflammatory cytokine levels that promote vascular leak, such as VEGF, which is the key mediator in AF progression to late wet AMD [3, 38, 39]. Higher mean serum VEGF levels were more prevalent in patients with wet AMD than in healthy controls, which could potentially enhance AF development by triggering acute intercalated disk remodelling [40, 41]. However, individuals with wet AMD frequently undergo long-term intravitreal injections of a VEGF inhibitor, a highly effective therapy for preserving and enhancing visual acuity. Notably, these inhibitors can penetrate the blood-retinal barrier, enter the systemic circulation, and potentially influence circulating VEGF levels in serum [42, 43]. This finding could partially elucidate the reduced trend observed in AF development among patients with wet AMD. Further studies are required to elucidate the underlying mechanisms.

In the sex-stratified analyses, we observed an association between AF and AMD among male patients but not among female patients. Firstly, it is well known that male patients generally have higher age-adjusted incidence and prevalence of AF than female patients [44, 45]. Consistent with previous epidemiologic studies [46, 47], the incidence of AF was slightly higher in male patients than in female patients in our analyses. In addition, some evidence suggests that male patients may have a higher prevalence of certain traditional CVDs risk factors, including hypertension [48], type 2 diabetes mellitus [49], and smoking [50], which could potentially contribute to the development of both conditions. Secondly, oestrogen and hormone replacement therapy in postmenopausal women, may play a significant role in supporting ocular function, particularly with respect to the retina [51]. As suggested by previous investigators, oestrogen/oestrogen receptors are found in ocular tissue and are involved in the regulation of the balance between cell survival, proliferation, and apoptosis [52, 53]. In addition, the protective effects of hormones help reduce atrial fibrosis, regulate ion channels, and maintain longer action potential durations, providing antiarrhythmic effects [54]. Therefore, the presence of sex hormones in female patients may, at least in part, account for the lack of a significant association between AF and AMD observed in our study, though, it should be considered that a substantial proportion of the female participants in our study were likely postmenopausal. Finally, the interaction of sex with the complement system, a related factor in the pathogenesis of AMD and AF, may explain our findings [55, 56]. However, the precise mechanism that underlies sex-differential associations was beyond the scope of our investigation.

One notable advantage of our study is its large-scale nationwide analysis using real-world data derived from routine clinical practice. However, this study is subject to some limitations. First, it was dependent on an administrative database that lacked comprehensive information regarding clinical characteristics such as lifestyle, smoking, body mass index, physical activity, and laboratory results. Although we employed propensity score methods, including stabilised IPTW and PSM, to adjust for several important parameters associated with clinical outcomes, bias from unmeasured confounders may still exist. Second, the ICD codes were used to define the outcomes rather than direct patient assessments. While previous studies [24–27] have employed or validated these codes, several patients with significant outcomes may not have been identified in the claims database, even if they did not actively seek medical assistance. Conversely, the underreporting of outcome events is expected to occur non-differentially across each treatment cohort, resulting in bias towards the null in the effect estimates [57, 58]. Lastly, the prevalence of AMD and AF in our investigation was greater than that reported in a previous general population study in other countries, potentially attributed to variations in study population, design, or healthcare systems [44, 59]. Consequently, further assessment is required to determine the generalisability of our findings to other ethnicities, nations, or demographics, and studies comprising multi-ethnic cohorts are necessary to confirm whether these findings are applicable to other races and geographic regions.

In conclusion, these data support the existence of a mutual relationship between AF and AMD, especially dry-type AMD. While the association between AMD and AF isn’t entirely unexpected, as both share age as a common factor, these findings underscore the significance of collaborative efforts between ophthalmologists and cardiologists in the managements of patients with AMD or AF. Early stages of AMD and AF may not exhibit symptomatology; hence, it is crucial for clinicians to proactively inquire and conduct thorough examinations to enable the early detection of AF or AMD. Further research is warranted to elucidate the pathophysiology of AF and AMD.

### Data and materials

The datasets generated or analysed during the current study are not publicly available because of the data protection policy of the NHIRD (https://nhird.nhri.org.tw/en/Data_Protection.html) but are available from the corresponding authors upon reasonable request.

## Summary

### What was known before

- Atrial fibrillation (AF) and age-related macular degeneration (AMD) share pathomechanisms involving chronic inflammation, oxidative stress, and vascular endothelial growth factor.
- However, the association between AMD and AF remains unknown.

### What this study adds

- By employing propensity score methods, such as propensity score matching and inverse probability of treatment weighting, to control for confounding variables, our data substantiates the presence of a reciprocal relationship between AF and AMD, particularly dry-type AMD, over a 20-year longitudinal national cohort study.

## Supplementary information

Supplementary Material
