# Supplementary material for: Association between atrial fibrillation and age-related macular degeneration: A nationwide cohort study
Source: Eye (Lond). 2025 Sep 5;39(15):2800–7. doi: 10.1038/s41433-025-03956-2 (PMC12494911; doi:10.1038/s41433-025-03956-2)

**Supplementary Material**

1. **Supplemental Tables**

**Table S1.** Diagnostic codes for comorbidities and anatomical therapeutic chemical codes for co-medication

**Table S2.** Demographic data of the original study population

**Table S3.** Demographic data of the study population after propensity score matching

**Table S4.** Association between AF and AMD after propensity score matching

1. **Supplemental Figure**

**Figure S1.** Cumulative incidence curves for outcomes (A) AMD and the risk of AF and

(B) AF and the risk of AMD.

**Table S1.** Diagnostic codes for comorbidities and anatomical therapeutic chemical codes for co-medication

| Baseline comorbidities | | |
| --- | --- | --- |
|  | ICD 9 | ICD 10 |
| Hypertension | 401-405 | I10-I16 |
| Diabetes mellitus | 250 | E08-E13 |
| Stroke | 430-438 | I60-69 |
| Heart failure | 428 | I50 |
| Coronary artery disease | 410-414 | I20-25 |
| Cirrhosis | 571.2, 571.5, 571.6 | K70.2, K70.3, K74 |
| COPD | 490-492, 494, 496 | J40-J44, J46 |
| Chronic kidney disease | 585 | N18 |
| Hyperlipidaemia | 272 | E78.0-78.5 |
| Malignancy | 140-208, 209.0-209.3 | C00-C96 |
| Obesity | 278, 649.2, 783.1, V45.86, V77.8, V85.2-V85.4 | E65-E68, R63.5, Z13.89 |
| Cataract | 366 | H25, H26, H28 |
| Baseline medication use | | |
|  | ATC code | |
| ACEI/ARB | C09A, C09B, C09C, C09D | |
| Beta blocking agents | C07 | |
| Calcium channel blockers | C08C, C08D | |
| Metformin | A10BA02 | |
| Statins | C10AA | |
| Fibrates | C10AB | |
| Antiplatelet | B01AC | |

Abbreviation: Angiotensin converting enzyme inhibitor, ACEI; Atrial fibrillation, AF; Age-related macular degeneration, AMD; ARB, [Angiotensin II receptor blockers](https://atcddd.fhi.no/atc_ddd_index/?code=C09C&showdescription=yes); COPD, Chronic obstructive pulmonary disease

**Table S2.** Demographic data of the original study population

| Characteristics† | AMD and the risk of AF | | |  | AF and the risk of AMD | | |
| --- | --- | --- | --- | --- | --- | --- | --- |
|  | **AMD** | | |  | **AF** | | |
|  | **AMD**  **(n = 31,766)** | **Non-AMD**  **(n = 31,766)** | **SMD**^‡^ |  | **AF**  **(n = 34,236)** | **Non-AF**  **(n = 34,236)** | **SMD**^‡^ |
| Mean age (SD), years | 69.87 (9.73) | 69.87 (9.73) | <0.001 |  | 73.26 (10.71) | 73.26 (10.71) | <0.001 |
| Sex, % |  |  | <0.001 |  |  |  | <0.001 |
| Male | 47.35 | 47.35 |  |  | 54.69 | 54.69 |  |
| Female | 52.65 | 52.65 |  |  | 45.31 | 45.31 |  |
| Income level (NTD), % |  |  |  |  |  |  |  |
| <15840 | 18.74 | 17.95 | 0.020 |  | 20.13 | 20.96 | 0.021 |
| 15,840-24,999 | 53.96 | 57.28 | 0.067 |  | 57.33 | 54.51 | 0.057 |
| 25,000-39,999 | 13.78 | 13.05 | 0.021 |  | 11.58 | 12.67 | 0.033 |
| ≥40,000 | 13.52 | 11.73 | 0.054 |  | 10.96 | 11.86 | 0.028 |
| CCI, mean (SD) | 1.19 (1.78) | 0.92 (1.60) | 0.157 |  | 1.71 (2.29) | 0.88 (1.61) | 0.418 |
| Comorbidities, % |  |  |  |  |  |  |  |
| Hypertension | 34.00 | 30.10 | 0.084 |  | 41.02 | 28.69 | 0.261 |
| Diabetes mellitus | 20.47 | 15.62 | 0.126 |  | 19.31 | 14.08 | 0.141 |
| Stroke | 7.30 | 6.61 | 0.027 |  | 16.73 | 7.14 | 0.299 |
| Heart failure | 2.45 | 1.98 | 0.032 |  | 18.40 | 2.27 | 0.550 |
| Coronary artery disease | 12.03 | 9.04 | 0.098 |  | 23.98 | 8.96 | 0.414 |
| Cirrhosis | 0.24 | 0.22 | 0.004 |  | 1.22 | 0.70 | 0.053 |
| COPD | 7.92 | 6.18 | 0.068 |  | 13.99 | 7.06 | 0.227 |
| Chronic kidney disease | 3.82 | 2.97 | 0.047 |  | 7.13 | 3.07 | 0.185 |
| Hyperlipidaemia | 22.60 | 17.52 | 0.127 |  | 17.91 | 14.68 | 0.088 |
| Malignancy | 5.26 | 4.54 | 0.033 |  | 6.27 | 4.23 | 0.092 |
| Obesity | 0.24 | 0.22 | 0.004 |  | 0.27 | 0.15 | 0.026 |
| Cataract | 46.19 | 11.16 | 0.840 |  | 10.63 | 9.92 | 0.023 |
| Other concomitant drugs, % | |  |  |  |  |  |  |
| ACEI/ARB | 33.41 | 27.81 | 0.122 |  | 42.22 | 28.46 | 0.291 |
| Beta blocking agents | 24.16 | 19.51 | 0.113 |  | 35.06 | 19.10 | 0.365 |
| Calcium channel blockers | 33.65 | 28.86 | 0.103 |  | 42.93 | 30.60 | 0.258 |
| Metformin | 16.45 | 11.78 | 0.134 |  | 12.75 | 11.22 | 0.047 |
| Statins | 21.15 | 16.20 | 0.127 |  | 16.09 | 14.20 | 0.053 |
| Fibrates | 4.16 | 3.31 | 0.045 |  | 3.65 | 3.10 | 0.030 |
| Antiplatelets | 26.93 | 21.63 | 0.124 |  | 41.70 | 23.95 | 0.385 |

Data are expressed as percentages unless otherwise indicated.

**^†^**All covariates listed were used to calculate the propensity score for analyses

^‡^A standardized mean difference of <0.1 indicates a negligible difference.

Abbreviation: Angiotensin converting enzyme inhibitor, ACEI; Atrial fibrillation, AF; Age-related macular degeneration, AMD; ARB, [Angiotensin II receptor blockers](https://atcddd.fhi.no/atc_ddd_index/?code=C09C&showdescription=yes); CCI, Charlson comorbidity index; COPD, Chronic obstructive pulmonary disease; NTD, New Taiwan Dollar; SD, Standard deviation; SMD, Standardized mean difference.

**Table S3.** Demographic data of the study population after propensity score matching

| Characteristics† | AMD and the risk of AF | | |  | AF and the risk of AMD | | |
| --- | --- | --- | --- | --- | --- | --- | --- |
|  | **AMD** | | |  | **AF** | | |
|  | **AMD**  **(n = 20,042)** | **Non-AMD**  **(n = 20,042)** | **SMD**^‡^ |  | **AF**  **(n = 22,770)** | **Non-AF**  **(n = 22,770)** | **SMD**^‡^ |
| Mean age (SD), years | 69.56 (10.13) | 69.89 (9.64) | 0.033 |  | 73.12 (10.68) | 73.73 (10.54) | 0.057 |
| Sex, % |  |  | 0.014 |  |  |  | 0.009 |
| Male | 47.58 | 46.86 |  |  | 55.16 | 54.73 |  |
| Female | 52.42 | 53.14 |  |  | 44.84 | 45.27 |  |
| Income level (NTD), % |  |  |  |  |  |  |  |
| <15840 | 20.33 | 18.13 | 0.056 |  | 20.12 | 21.22 | 0.027 |
| 15,840-24,999 | 52.70 | 55.74 | 0.061 |  | 56.91 | 55.06 | 0.037 |
| 25,000-39,999 | 13.64 | 13.87 | 0.007 |  | 11.72 | 12.38 | 0.020 |
| ≥40,000 | 13.33 | 12.26 | 0.032 |  | 11.25 | 11.34 | 0.003 |
| CCI, mean (SD) | 0.91 (1.64) | 0.91 (1.59) | <0.001 |  | 1.09 (1.80) | 1.11 (1.81) | 0.013 |
| Comorbidities, % |  |  |  |  |  |  |  |
| Hypertension | 25.94 | 26.53 | 0.013 |  | 31.96 | 33.68 | 0.037 |
| Diabetes mellitus | 15.91 | 15.95 | 0.001 |  | 14.84 | 15.49 | 0.018 |
| Stroke | 5.55 | 5.85 | 0.013 |  | 10.62 | 10.61 | 0.000 |
| Heart failure | 1.89 | 2.01 | 0.009 |  | 4.55 | 3.42 | 0.058 |
| Coronary artery disease | 8.87 | 9.30 | 0.015 |  | 13.63 | 13.25 | 0.011 |
| Cirrhosis | 0.71 | 0.70 | 0.001 |  | 0.93 | 0.93 | 0.000 |
| COPD | 6.17 | 6.22 | 0.002 |  | 9.13 | 9.34 | 0.007 |
| Chronic kidney disease | 2.79 | 2.93 | 0.008 |  | 4.24 | 4.21 | 0.001 |
| Hyperlipidaemia | 17.28 | 17.23 | 0.001 |  | 15.18 | 15.80 | 0.017 |
| Malignancy | 4.02 | 4.05 | 0.002 |  | 4.97 | 5.19 | 0.010 |
| Obesity | 0.21 | 0.21 | <0.001 |  | 0.21 | 0.21 | <0.001 |
| Cataract | 17.88 | 17.69 | 0.005 |  | 9.45 | 9.52 | 0.002 |
| Other concomitant drugs, % | |  |  |  |  |  |  |
| ACEI/ARB | 30.48 | 31.13 | 0.014 |  | 34.57 | 37.06 | 0.052 |
| Beta blocking agents | 22.65 | 23.61 | 0.023 |  | 26.04 | 27.65 | 0.036 |
| Calcium channel blockers | 31.93 | 33.35 | 0.030 |  | 37.07 | 40.10 | 0.062 |
| Metformin | 15.19 | 14.87 | 0.009 |  | 11.83 | 12.67 | 0.026 |
| Statins | 18.16 | 18.41 | 0.006 |  | 14.66 | 15.55 | 0.025 |
| Fibrates | 3.84 | 3.89 | 0.003 |  | 3.42 | 3.59 | 0.009 |
| Antiplatelets | 25.15 | 26.08 | 0.021 |  | 32.45 | 34.45 | 0.042 |

Data are expressed as percentages unless otherwise indicated.

**^†^**All covariates listed were used to calculate the propensity score for analyses

^‡^A standardized mean difference of <0.1 indicates a negligible difference.

Abbreviation: Angiotensin converting enzyme inhibitor, ACEI; Atrial fibrillation, AF; Age-related macular degeneration, AMD; ARB, [Angiotensin II receptor blockers](https://atcddd.fhi.no/atc_ddd_index/?code=C09C&showdescription=yes); CCI, Charlson comorbidity index; COPD, Chronic obstructive pulmonary disease; NTD, New Taiwan Dollar; SD, Standard deviation; SMD, Standardized mean difference.

**Table S4.** Association between AMD and AF after propensity score matching

| Comparison/Outcomes | Patients, n | Events, n | Person-years at risk | Incidence rate^†^ | HR^‡^ | 95% CI | *p* value |
| --- | --- | --- | --- | --- | --- | --- | --- |
| AMD and the risk of AF | | | | | | | |
| AMD cohort | 20042 | 1546 | 157539 | 9.81 | 1.08 | 1.01–1.17 | 0.034 |
| Non-AMD cohort | 20042 | 1246 | 140161 | 8.89 | 1.00 | Reference |  |
| AF and the risk of AMD | | | | | | | |
| AF cohort | 22770 | 1439 | 119790 | 12.01 | 1.09 | 1.02–1.17 | 0.014 |
| Non-AF cohort | 22770 | 1598 | 145306 | 11.00 | 1.00 | Reference |  |

^†^Per 1000 person-years

^‡^The hazard ratio was calculated by a univariable Cox regression model after propensity score matching.

Abbreviation: AF, atrial fibrillation; AMD, age-related macular degeneration; CI, confidence interval; HR, hazard ratio; SD, standard deviation.

**Figure S1.** Cumulative incidence curves for outcomes (A) AMD and the risk of AF and (B) AF and the risk of AMD. *AF*, Atrial fibrillation; *AMD*, Age-related macular degeneration.


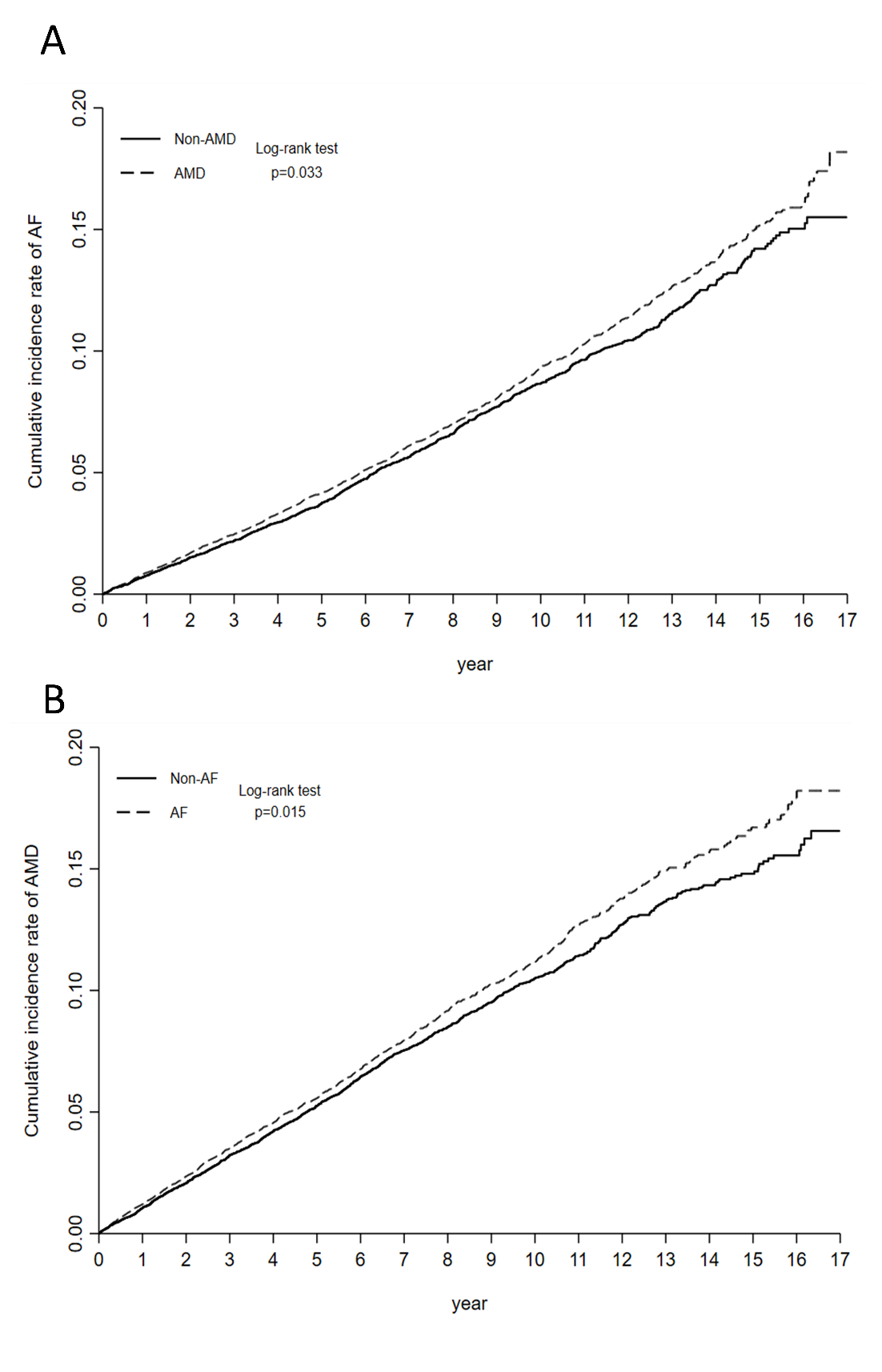

Supplement: Supplementary file 1 — Supplementary Material [file 41433_2025_3956_MOESM1_ESM.docx]
